# Mucosal Exosome Proteomics of Hybrid Grouper *Epinephelus fuscoguttatus*♀ × *E. lanceolatus*♂ Infected by *Pseudomonas plecoglossicida*

**DOI:** 10.3390/ani14233401

**Published:** 2024-11-25

**Authors:** Dong Yang, Xiaowan Ma, Shengping Zhong, Jiasen Guo, Dewei Cheng, Xuyang Chen, Teng Huang, Lixing Huang, Ying Qiao, Theerakamol Pengsakul

**Affiliations:** 1 Guangxi University, Nanning 530200, China; yangdong201602@163.com (D.Y.); bowl_88@hotmail.com (X.M.); tenghuang@163.com (T.H.); 2 Key Laboratory of Tropical Marine Ecosystem and Bioresource, Fourth Institute of Oceanography, Ministry of Natural Resources, Beihai 536000, China; guojiasen@4io.org.cn (J.G.); chengdewei@4io.org.cn (D.C.); chenxuyang@4io.org.cn (X.C.); 3 Institute of Marine Drugs, Guangxi University of Chinese Medicine, Nanning 530200, China; shpzhong@foxmail.com; 4 Fisheries College, Jimei University, Xiamen 361000, China; 5 Health and Environmental Research Center, Faculty of Environmental Management, Prince of Songkla University, Hat Yai 90110, Thailand; theerakamol.p@psu.ac.th

**Keywords:** *Pseudomonas plecoglossicida*, exosomes, proteomic analysis, biomarker

## Abstract

*Pseudomonas plecoglossicida* infection, which causes visceral white spot disease, is a significant and economically devastating disease in aquaculture. In this study, we investigated the impact of bacterial infection on the protein composition of exosomes derived from the surface mucus of the hybrid grouper *Epinephelus fuscoguttatus*♀ × *E. lanceolatus*♂. Two hundred healthy fish were randomly separated into challenge and control groups. Fish from the challenge group received 10^3^ CFU/g of the bacterial pathogen *P. plecoglossicida* via intraperitoneal injection, while sterile PBS was used as a negative control. After injection, the mucus was collected and the exosomes were extracted for proteomic analysis. The results of proteomic analysis revealed that *P. plecoglossicida* infection significantly increased the levels of innate immune proteins, including lysosomal and peroxisomal proteins, within the exosomes. Furthermore, the CAD protein was found to play a pivotal role in the protein interaction networks involved in the response to *P. plecoglossicida* infection. Intriguingly, we also observed a significant increase in the levels of metal-binding proteins within the exosomes, providing important evidence of nutritional immunity on the surfaces of the fish hosts. Notably, several proteins, such as plasma kallikrein, Annexin A5, eukaryotic translation initiation factor 3 subunit M, and S-methyl-5-thioadenosine phosphorylase, exhibited a remarkable increase in abundance in exosomes after infection. These proteins show promising potential as noninvasive biomarkers for the diagnosis of visceral white spot disease. The study contributes to the understanding of the host response to *P. plecoglossicida* infection and may aid policymakers in implementing appropriate intervention measures for effective risk management of this devastating disease.

## Simple Summary

*Pseudomonas plecoglossicida* infection, which causes visceral white spot disease, is a significant and economically devastating disease in aquaculture. In this study, we investigated the impact of bacterial infection on the protein composition of exosomes derived from the surface mucus of the hybrid grouper *Epinephelus fuscoguttatus*♀ × *E. lanceolatus*♂, and studied the host response to *P. plecoglossicida* infection and potential noninvasive biomarkers for the diagnosis of visceral white spot disease.

## 1. Introduction

Infectious disease has become one of the significant limiting factors for the healthy development of aquaculture in China. Visceral white spot disease caused by *Pseudomonas plecoglossicida* is a prevalent bacterial disease in large yellow croaker aquaculture, resulting in significant economic losses, with mortality rates reaching 80% [1,2]. Visceral white spot disease primarily occurs in spring and autumn, resulting in a covert initial phase without visible symptoms on the body surface of the fish. The diseased fish have white nodules of varying sizes from 0.5 to 3 mm in the internal organs such as liver, spleen, kidney, etc., hence the name "visceral white spot disease" [3]. At present, *P. plecoglossicida* is widely recognized as the pathogen of visceral white spot disease in large yellow croaker [3,4].

*Pseudomonas plecoglossicida* is an aerobic Gram-negative bacillus with polar flagella. Diseases caused by *P. plecoglossicida* in aquaculture animals have been reported in large yellow croakers [5,6], sweetfish [7], rainbow trout [8], etc., resulting in huge economic losses. In addition, previous studies found that artificial infection with *P. plecoglossicida* could also cause "visceral white spot disease" in grouper [9]. Given the great harm of *P. plecoglossicida* to the aquaculture industry, its pathogenic mechanism has attracted wide attention. Existing studies have shown that *P. plecoglossicida* possesses various virulence factors such as type III secretion system, type VI secretion system [10], and cytotoxin ExoU [3], which can survive and proliferate within macrophages [4], and its pathogenicity is significantly temperature-dependent [11]. These findings provide valuable basic data for further research on the pathogenic mechanisms of *P. plecoglossicida*. But conventional drug treatments become ineffective in later stages of visceral white spot disease when infected fish experience feeding difficulties during the aquaculture process. Therefore, noninvasive diagnostic techniques targeting *P. plecoglossicida* infections are crucial for timely intervention and effective risk management of visceral white spot disease, providing valuable insights for policymakers and stakeholders in preventing and controlling this devastating disease.

However, the limitations of such traditional pathogen diagnosis methods still exist and hinder the development and deployment of pathogen early warning systems, posing significant challenges to achieving true outbreak monitoring and risk management [12,13]. Therefore, there is an urgent need to develop noninvasive detection technologies for major aquaculture diseases; monitor the introduction, transmission, and persistence of pathogens in the process of aquaculture; and propose key control points to effectively interrupt pathogen spread [14,15]. These findings provide important theoretical references for decision-makers and fish farmers.

Exosomes, which serve as essential mediators of intercellular communication, contain various biological molecules, including proteins, nucleic acids, and lipids [16,17]. Considering their unique cargo content, ubiquitous presence in various biological fluids, high biological stability, and intercellular communication functionality, exploring the utility of exosomes as biomarkers in liquid biopsies is inevitable [18,19]. Furthermore, the impact of exosomes in certain diseases further supports their potential as biomarkers [20].

Characterizing the proteomic profile of exosomes can provide insights into identifying amplified signaling pathways indicative of disease [21,22]. The diagnostic potential of exosomes extends beyond tissues and organs [23,24,25]. In teleost, the skin plays a crucial role in anti-infective immunity and acts as a frontline barrier through the secretion of epidermal mucus [14]. Notably, this mucus is also rich in exosomes [15]. Exploring the contents of exosomes, particularly proteins, within the mucus could provide insights into intercellular communication during infection within the fish skin mucosal immune system and contribute to the identification of disease-related biomarkers [26].

To date, the isolation of exosomes from fish has focused mainly on leukocyte culture medium from Atlantic salmon [27], plasma [28], semen [29], the mucus of Chinese tongue sole [30], the mucus of cod [31], and the plasma of nurse sharks [32], although research remains limited. Overall, an understanding of the composition of exosomes within fish mucus and their role in intercellular communication during infection has potential implications for the development of disease-specific biomarkers. Further investigations in this field could lead to advancements in our comprehension of the fish skin mucosal immune system and aid in disease diagnosis and management.

In this study, we focused on the infection of hybrid grouper (*Epinephelus fuscoguttatus*♀ × *E. lanceolatus*♂) with *P. plecoglossicida*, followed by the collection of fish surface mucus and extraction of exosomes for proteomic analysis. By investigating the innate immune strategies of mucus exosomes and identifying potential biomarkers for early noninvasive diagnosis, this study aims to contribute to the overall health and sustainability of grouper aquaculture.

## 2. Materials and Methods

### 2.1. Sample Collection and Exosome Isolation

A total of 200 healthy hybrid groupers (*Epinephelus fuscoguttatus*♀ × *E. lanceolatus*♂) weighing 71.39 ± 6.11 g and 16 ± 0.72 cm in length were obtained from an aquaculture farm in Beihai, Guangxi, China. All fish were cultured and acclimatized at 18 °C for 15 days using flowing sand filtered seawater in a 200 L tank. The fish were fed with commercial feed during the acclimation process. All fish were tested to be healthy by sera agglutination and bacteriological recovery tests as described by Qi et al. [33]. The fish were randomly separated into two experimental groups (challenge group and control group). Each fish received 10^3^ CFU/g of the bacterial pathogen *P. plecoglossicida* via intraperitoneal injection, while sterile PBS was used as a negative control. The *P. plecoglossicida* strain used in the present study was originally isolated and identified from afflicted groupers from Guangxi Province. The injection dosage (LD_50_) was determined according to our previous experiment and aligned with the findings reported by Qi et al. [33]. Three days after injection, the vitality and swimming speed of the surviving groupers decreased. The surviving groupers were dissected and dense white nodules in their internal organs (liver, spleen, and kidneys) were observed, which is a typical symptom of visceral white spot disease. After being anesthetized by MS-222 (Sigma Aldrich, St. Louis, MO, USA), epidermal mucus was collected via scraping the fish skin using a sterilized glass slide. The mucus was immediately frozen in liquid nitrogen and stored at −80 °C for further study. For exosome isolation, the collected mucus was diluted with sterilized PBS solution and filtered using a 0.45 mm hydrophilic filter membrane. The exosomes were then isolated from the filtrate using Total Exosome Isolation Kit (4484453, Invitrogen™, Shanghai, China) according to the manufacture’s guide.

### 2.2. Exosomes Characterization

#### 2.2.1. Morphology Characterization Using Transmission Electron Microscopy (TEM)

Prepared exosome samples were pipetted onto copper grids and naturally adsorbed for 5–10 min. Excess liquid was removed and the grids were air-dried at room temperature. A 20 μL drop of 2% phosphotungstic acid was then added onto the copper grids, followed by a 3–5 min incubation. Exosomes were observed and photographed under an HT7700 transmission electron microscope (HITACHI, Tokyo, Japan) [30].

#### 2.2.2. Particle Size Measurement Using Nanoparticle Tracking Analysis (NTA)

The obtained exosome pellet was resuspended in 200 μL DEPC PBS solution and transferred to 1.5 mL EP tubes for NTA experiments. Briefly, 20 μL of PBS-exosomes was diluted 40-fold with PBS and analyzed using the NS300 nanoparticle size analyzer (Malvern Instruments Ltd., Malvern City, UK). Particle size of the exosomes was calculated on a particle-by-particle basis in a 60 s video recorded at a frame rate of 25 frames per second and then results were analyzed using NTA software version 3.2 [34].

#### 2.2.3. Western Blot Analysis

Western blot analysis was conducted according to Kowal [35] with minor revision. Protein samples of exosomes were prepared using RIPA lysis buffer (P0013B, Beyotime, Beijing, China) and boiled at 100 °C for 5–6 min after mixing with loading buffer. SDS-PAGE was performed to separate the proteins, followed by transfer onto PVDF membranes (IPVH00010, Millipore, MA, USA). The membranes were blocked with 5% skim milk (0.75 g powdered milk + 15 mL PBS) at 37 °C for 1–2 h. After washing with 1× PBST (1000 mL 1× PBS + 1 mL Tween-20) three times, the membranes were incubated with primary antibodies (CD81 antibody (66866-1-Ig): 1:500; CD63 antibody (25682-1-AP): 1:1000; HSP70 antibody (10995-1-AP): 1:2000; TSG101 antibody (28283-1-AP): 1:1000; GAPDH antibody (D110016-0200): 1:10,000; PROTEINTECH, USA) overnight at 4 °C. Following three washes with 1× PBST, the membranes were incubated with secondary antibodies (rabbit secondary antibody (111-035-003): 1:5000; Jackson ImmunoResearch, Pennsylvania, PA, USA) at 37 °C for 1 h. After three final washes with 1× PBST, chemiluminescent detection was performed using a Chemiluminescence Imaging System (4600 Chemiluminescence Analyzer, Tanon Science & Technology Co., Ltd., Shanghai, China), and the results were photographed and recorded.

### 2.3. Exosomal Protein Analysis

#### 2.3.1. Total Protein Extraction and Protein Quality Test

The obtained exosomes were thawed and centrifuged at 14,000× *g* for 15 min. After discarding the permeate, the ultra-filtered sample was put into a 1.5 mL centrifuge tube and balanced with DB buffer (8 M Urea, 100 mM TEAB, pH 8.5). The supernatant was taken and 1 M DTT was added to react for 1 h at 56 °C, and subsequently alkylated with sufficient IAM for 1 h in the dark at room temperature.

The Bradford protein quantitative kit was used for the protein quality test of the exosomes according to the manufacturer’s instructions. Then 20 μg of the samples were loaded to 12% SDS-PAGE gel electrophoresis, wherein the concentrated gel was performed at 80 V for 20 min, following the separation gel at 120 V for 90 min. The gel was stained using Coomassie brilliant blue R-250 and decolored with destaining solution.

#### 2.3.2. Trypsin Digestion and Fraction Separation

The trypsin digestion and fraction separation were conducted according to Choi [36]. Briefly, the volume of each protein sample was made up to 100 μL with DB lysis buffer (8 M Urea, 100 mM TEAB, pH 8.5), trypsin and 100 mM TEAB buffer were added, sample was mixed and incubated at 37 °C for 4 h. Then trypsin and CaCl_2_ were added and digested overnight. After digestion, formic acid was added to adjust pH lower to 3, then sample was centrifuged at 12,000× *g* for 5 min at room temperature. The supernatant was collected and slowly loaded to the C18 desalting column, washed three times with washing buffer (0.1% formic acid, 3% acetonitrile), and then eluted using elution buffer (0.1% formic acid, 70% acetonitrile). The eluent was collected and lyophilized.

The gradient elution was conducted using mobile phase A (2% acetonitrile, pH 10.0) and mobile phase B (98% acetonitrile, pH 10.0). The lyophilized powder was dissolved in mobile phase A and centrifuged at 12,000× *g* for 10 min at room temperature. The sample was fractionated using a C18 column (Waters BEH C18, 4.6 × 250 mm, 5 μm) on a Rigol L3000 HPLC system platform at 45 °C. Details of elution gradient are listed in Supplementary Table S1. The eluates were monitored at UV 214 nm, collected to a sterilized tube per minute. All fractions were dried under vacuum, and then, reconstituted in 0.1% (*v*/*v*) FA (formic acid) in water.

#### 2.3.3. LC-MS/MS Analysis

Proteomic analysis was conducted by Novogene Co., Ltd. (Beijing, China) using EASY-nLCTM 1200 UHPLC system (Thermo Fisher, MA, USA) coupled with Q ExactiveTM HF-X mass spectrometer (Thermo Fisher, MA, USA) [37]. Briefly, the lyophilized powder was dissolved in 10 μL of mobile phase A (100% water, 0.1% formic acid), centrifuged at 14,000× *g* for 20 min at 4 °C, and 1 μg of the supernatant was injected into a home-made C18 Nano-Trap column (4.5 cm × 75 μm, 3 μm). The temperature of the column oven was set to 55 °C. Peptides were separated in a home-made analytical column (15 cm × 150 μm, 1.9 μm), using a linear gradient elution as listed in Supplementary Table S1. The separated peptides were analyzed by Q ExactiveTM HF-X mass spectrometer, with ion source of Nanospray FlexTM (ESI), spray voltage of 2.1 kV and ion transport capillary temperature of 320 °C. Full scan ranges from *m*/*z* 350 to 1500 with resolution of 60,000 (at *m*/*z* 200), an automatic gain control (AGC) target value was 3 × 106 and the maximum ion injection time was 20 ms. The top 40 precursors of the highest abundance in the full scan were selected and fragmented by higher energy collisional dissociation (HCD) and analyzed in MS/MS, where resolution was 15,000 (at *m*/*z* 200), the automatic gain control (AGC) target value was 1 × 105, the maximum ion injection time was 45 ms, a normalized collision energy was set as 27%, an intensity threshold was 2.2 × 10^4^, and the dynamic exclusion parameter was 20 s.

#### 2.3.4. Proteomics Bioinformatics Analysis

The proteins with FC > 2.0 or FC < 0.5 (FC, fold change, *p* < 0.05) were defined as differentially expressed proteins (DEP). Proteins with cellular component (CC), biological process (BP), and molecular function (MF) functions were annotated using GeneOntology database (www.geneontology.org). The Kyoto Encyclopedia of Genes and Genomes (KEGG) database (http://www.genome.jp/kegg/) was used to perform the pathway analysis. The GSEA analysis was conducted using https://www.gsea-msigdb.org/gsea/index.jsp. The protein-protein interactions were predicted using the STRING-db server (http://string.embl.de/, accessed on 28 August 2024.).

### 2.4. Biomarkers Screen and Validation

Proteins exhibiting a remarkable increase in exosomes after *P. plecoglossicida* infection were screened and selected as potential biomarkers. Skins from both control group and challenge group of the groupers were collected and validated for the expression of thes candidate biomarkers using qRT-PCR [38]. β-actin was selected as the housekeeping reference gene [39] and the primers used in the qRT-PCR assays are listed in Table 1.

## 3. Results

### 3.1. Mucous Exosomes Characterization

**Figure 1:**
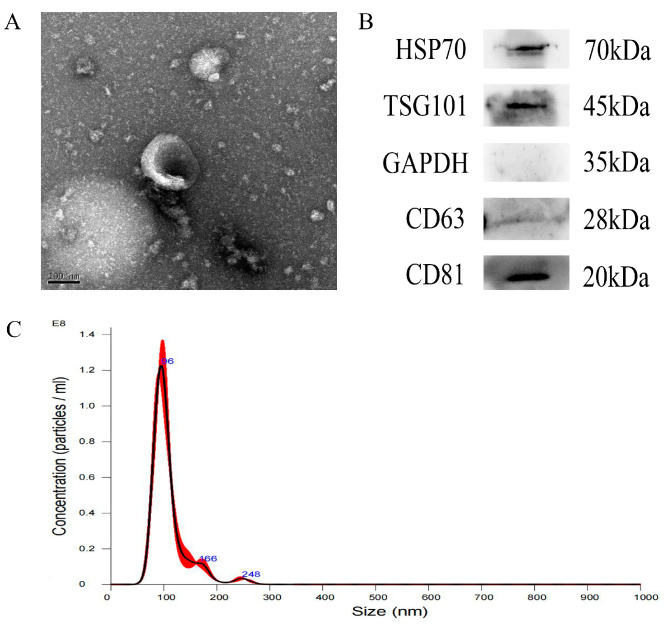
Characterization of exosomes from the mucus of the hybrid grouper *Epinephelus fuscoguttatus*♀ × *E. lanceolatus*♂. (**A**) Transmission electron microscopy observation of exosomes. Field of view = 100 nm; (**B**) Western blotting of exosomes using HSP70, TSG101, CD81, CD63 and GAPDH; (**C**) NTA analysis of exosomes showing the particle size distributions and concentrations.

By collecting mucus from the surface of groupers and subjecting it to ultracentrifugation, we obtained exosomes from the mucus. Subsequently, we characterized and identified the exosomes using techniques such as transmission electron microscopy (TEM), western blot (WB), and nanoparticle tracking analysis (NTA). The isolation process successfully yielded exosomes with typical ultrastructure and particle size, as demonstrated by TEM (Figure 1). TEM images revealed the presence of saucer-like particles that were either gathered or scattered in the field of view (Figure 1A). WB analysis confirmed the exosomal nature of the samples by detecting the presence of CD63, CD81, GAPDH, HSP70, and TSG101 proteins (Figure 1B). NTA results showed that the exosome particles had an average diameter of 108.8 nm, with the main peak observed at 95.0 nm (Figure 1C). The concentration of exosomes in the samples was approximately 5.5 × 10^9^ particles/mL.

### 3.2. Quality Control of Proteomic Sequencing

**Figure 2:**
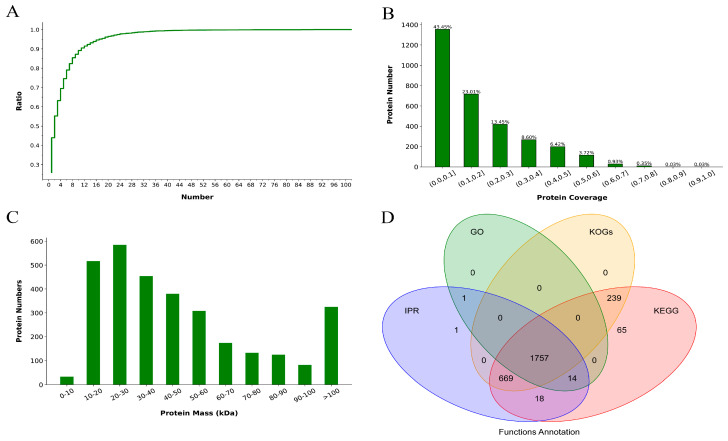
Quality control of proteomic sequencing results. (**A**) Distribution of unique peptides; (**B**) protein coverage distribution, where the *x*-axis represents the coverage range and the *y*-axis represents the number of proteins in each corresponding range; (**C**) distribution of protein molecular weights, where the *x*-axis represents the identified protein molecular weights and the *y*-axis represents the number of identified proteins; (**D**) functional annotation analysis via the IPR, GO, KOG, and KEGG databases.

In this study, we employed label-free quantitative proteomic analysis to investigate the protein cargo inside the mucus exosomes. A total of 3116 proteins were identified in the two samples, among which 2776 proteins were quantified. We conducted a series of quality controls on the protein sequencing results, including analysis of the distribution of unique peptide numbers, protein coverage, and protein molecular weight. The distribution of unique peptides is shown in Figure 2A, with the *x*-axis representing the number of unique peptides and the *y*-axis representing the cumulative percentage of proteins with the corresponding number of unique peptides. A flatter curve indicates a higher number of unique peptides and more reliable protein identification. Figure 2B displays the protein coverage distribution, with the *x*-axis representing the coverage range (the ratio of the length of detected peptides covering a protein to the total length of the protein) and the *y*-axis representing the number of proteins in each corresponding range. Figure 2C shows the distribution of protein molecular weights, with the *x*-axis representing the identified protein molecular weights (in kilodaltons, kDa) and the *y*-axis representing the number of identified proteins. The distribution of protein molecular weights is an important indicator for evaluating the size of identified proteins. A wider range of molecular weights indicates a broader range of identified proteins. Identified credible proteins exhibited a broad mass distribution, with the majority of proteins under 100 kDa. Additionally, annotation analysis using popular databases such as IPR, GO, KOGs, and KEGG revealed that 1757 proteins could be successfully annotated by all four databases (Figure 2D). Based on the comprehensive and high-quality protein data obtained through proteomic sequencing, we have laid a solid foundation for further analysis and exploration.

### 3.3. Functional Annotation of Protein Cargos Inside the Mucus Exosomes

**Figure 3:**
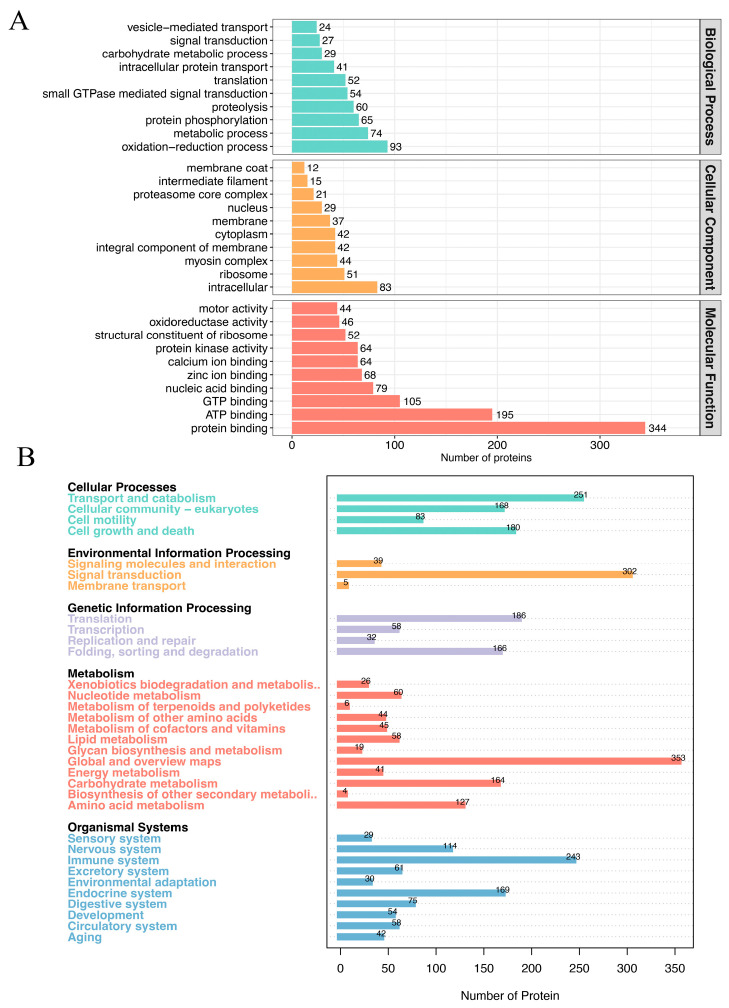
GO and KEGG enrichment analyses of the exosomal proteins. (**A**) GO enrichment analysis; Biological Process, Cellular Component and Molecular Function are indicated with cyan, orange and red color; (**B**) KEGG enrichment analysis. Cellular Processes, Environmental Information Processing, Genetic Information Processing, Metabolism and Organismal Systems are indicated with cyan, orange, lilac, red and blue color.

**Figure 4:**
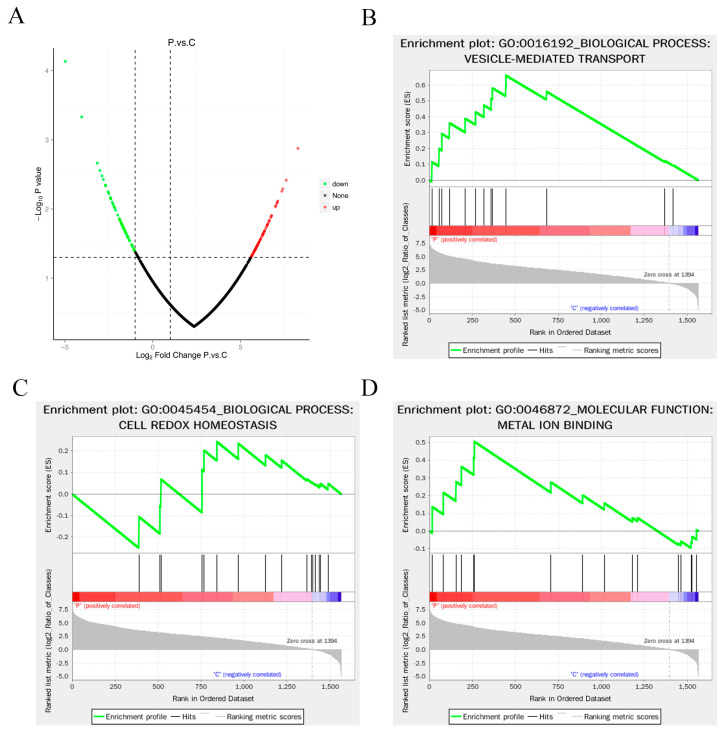
Enrichment analysis of differentially expressed proteins. (**A**) Volcano plot of differentially expressed proteins; (**B**–**D**) GSEA-GO enrichment analysis of DEPs.

Currently, there is insufficient understanding of the protein cargo carried by exosomes in the mucus of fish due to a lack of relevant research. To gain preliminary insights into the protein cargo composition of grouper mucus exosomes, we performed GO (Figure 3A) and KEGG (Figure 3B) enrichment analysis on the exosomal proteins. Figure 4A displays the results of the GO enrichment analysis, revealing that the majority of proteins were categorized under Molecular Function, with the highest proportions attributed to protein binding, ATP binding, and GTP binding. Additionally, 46 proteins were found to participate in oxidoreductase activity, highlighting the role of grouper mucus as an innate immune barrier. Interestingly, 64 proteins were associated with calcium ion binding, and 68 proteins were associated with zinc ion binding. Calcium and zinc are essential micronutrients for microbial survival. The host can block the proliferation of pathogens and the spread of infection by sequestering these micronutrients, a phenomenon referred to as nutritional immunity. While the presence of nutritional immunity in fish mucus has not been reported, this study may provide the important clue. Moreover, this underscores the significant role of mucus in the innate immune system of fish. Additionally, we identified several proteins closely related to vesicle-mediated transport, which may provide clues to understanding the formation and transport mechanisms of exosomes in mucus. Figure 3B shows the results of the KEGG enrichment analysis, indicating that the protein cargo was enriched in various pathways. Notably, 302 proteins were enriched in the “Signaling molecules and interaction” pathway, suggesting the important role of exosomal protein cargo in intercellular communication. Furthermore, 243 proteins were enriched in the “immune system” pathway, providing intriguing insights into the defense mechanisms of fish mucus against pathogens.

### 3.4. Enrichment Analysis of Differentially Expressed Proteins

In this study, we identified upregulated proteins based on Fold change > 2.0 and *p* value < 0.05, while downregulated proteins were identified based on Fold change < 0.50 and *p* value < 0.05. A total of 126 differentially expressed proteins were obtained, including 68 significantly upregulated and 58 significantly downregulated proteins (Supplementary Table S2 and Figure 4A). The top 20 upregulated and downregulated proteins are listed in Table 2, including extracellular proteins, cytoplasm proteins, plasma membrane proteins, nucleus proteins and centrosome proteins. Table 2 presents the top 20 upregulated and downregulated proteins, encompassing a broad range of subcellular localizations, such as extracellular, cytoplasmic, plasma membrane, nuclear, and centrosomal proteins. This widespread distribution of differentially expressed proteins signifies the intricate and diverse biological functions of mucus vesicles.

GO enrichment analysis classified the majority of differentially expressed proteins into functional categories related to biological processes, particularly primary metabolic process, cellular metabolic process, and protein metabolic process (Supplementary Figure S1). Further Gene Set Enrichment Analysis (GSEA) was performed on the selected GO enrichment results. Traditional protein function enrichment methods based on hypergeometric tests may yield limited or no results when individual protein expression changes are relatively small. GSEA analysis effectively compensates for the limitations of traditional enrichment analyses, providing comprehensive insights into the modulation of functional units such as pathways, GO terms, or others. The basic idea is to use predefined protein sets, rank the proteins based on their differential expressions in the two sample categories, and test whether the predefined protein sets are enriched at the top or bottom of this ranking table. Using GSEA-GO enrichment analysis, we discovered a positive correlation between differentially expressed proteins involved in vesicle-mediated transport and bacterial infection (Figure 4B). This suggests that bacterial infection may promote the secretion and transport of extracellular vesicles in fish mucus. Additionally, GSEA-GO enrichment analysis also indicated a close association between cell redox homeostasis (Figure 4C) and metal ion binding (Figure 4D) with differentially expressed proteins. Redox homeostasis represents the balance of oxidative and reducing reactions in all living systems. The differential expression of proteins related to oxidative-reduction reactions suggests that mucus vesicles may induce these reactions to clear surface pathogens and prevent further infection. It is noteworthy that most metal ion binding proteins in mucus vesicles were significantly upregulated following bacterial infection. This unexpected finding reveals the presence of nutritional immunity in fish mucus, as metal ion binding proteins reduce the availability of essential elements (e.g., iron, zinc, manganese, and nickel) for bacteria. These elements play crucial roles in biological processes such as precursor biosynthesis, DNA replication, transcription, respiration, and oxidative stress responses. This discovery represents an important report of nutritional immunity in fish mucus and provides valuable insights for understanding innate immunity in fish.

**Figure 5:**
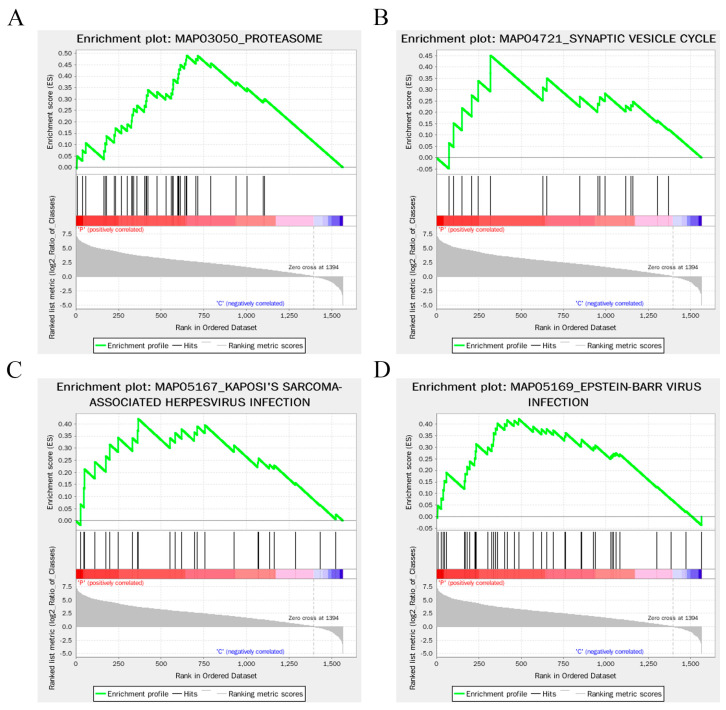
GSEA-KEGG enrichment analysis. (**A**–**D**) GSEA-KEGG enrichment analysis of DEPs.

KEGG enrichment analysis highlighted the enrichment of 126 differentially expressed proteins across multiple signaling pathways. Surprisingly, several pathways were found to be associated with cancer, including melanoma (Supplementary Figure S2). We speculated that these cancer cell clearance-associated proteins might also be involved in the clearance of pathogens. Further GSEA-KEGG enrichment analysis unveiled a significant upregulation of differentially expressed proteins associated with proteasome, synaptic vesicle cycle, Kaposi’s sarcoma-associated herpesvirus infection, and Epstein-Barr virus infection in mucus vesicles following bacterial infection (Figure 5A–D). This intriguing result not only reaffirms the crucial role of mucus vesicles in eliminating surface pathogens but also implies that vesicles may employ protein degradation mechanisms for pathogen clearance. Combining the aforementioned oxidative-reduction and metal ion shielding mechanisms, the mechanism underlying pathogen clearance by mucus vesicles appears to be more intricate than previously envisioned.

### 3.5. Subcellular Localization Analysis of Differentially Expressed Proteins

Formation of vesicles involves inward membrane invagination, followed by the formation of intraluminal vesicles which then fuse with the cell membrane, ultimately releasing as mucus vesicles into the extracellular space. The rate of vesicle generation differs across cell types, resulting in highly heterogeneous vesicle size and composition. Analyzing the subcellular localization of proteins within mucus vesicles offers valuable insights into their functional mechanisms. Thus, in this study, we performed an enrichment analysis of the subcellular localization of differentially expressed proteins in mucus vesicles to gain further understanding of their role in pathogen clearance.

**Figure 6:**
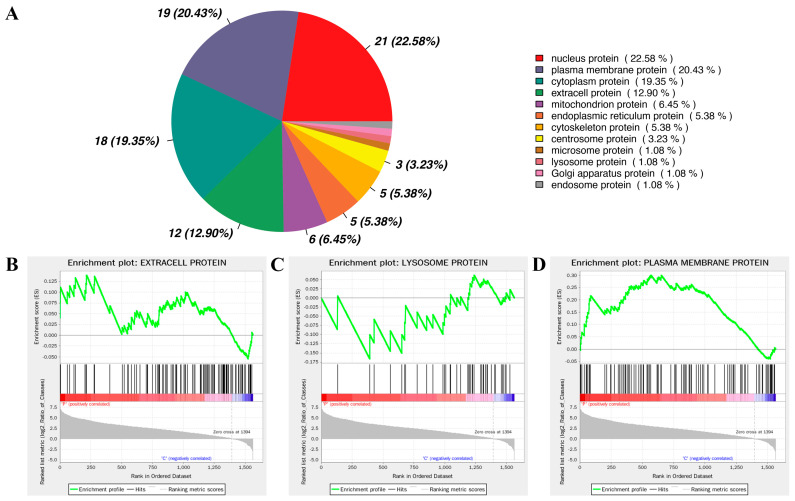
Subcellular localization analysis of DEPs. (**A**) Pie chart analysis revealed that the DEPs in mucus vesicles were nuclear proteins (22.58%), plasma membrane proteins (20.43%), cytoplasmic proteins (19.35%), and extracellular proteins (12.90%); (**B**–**D**) GSEA enrichment analysis.

Our analysis revealed that the most abundant differentially expressed proteins in mucus vesicles were nucleus proteins (22.58%), plasma membrane proteins (20.43%), cytoplasm proteins (19.35%), and extracellular proteins (12.90%) (Figure 6A). The high proportions of plasma membrane proteins, cytoplasm proteins, and extracellular proteins align with the process of mucus vesicle formation. However, the unexpectedly high proportion of nucleus proteins is intriguing. Nucleus proteins mainly participate in transcriptional regulation of gene expression, and their presence in mucus vesicles suggests a potential influence on gene expression in recipient skin cells upon fusion. This may subsequently modulate the response of skin cells to bacterial infection, indicating a complex mechanism that warrants further investigation.

Furthermore, GSEA enrichment analysis revealed a close correlation between differentially expressed proteins in mucus vesicles and extracellular proteins, lysosome proteins, and plasma membrane proteins (Figure 6B–D). This intriguing finding suggests that the protein composition on the surface of mucus vesicles undergoes significant changes following bacterial infection, potentially offering candidate biomarkers for diagnostic biomarker discovery. Moreover, it emphasizes the critical role of lysosome proteins in the process of pathogen clearance by mucus vesicles. The significantly altered plasma membrane proteins are highlighted in Table 2 (black bold letter), which includes Catenin alpha-1, Annexin A13, Coatomer subunit beta, Serine/threonine-protein kinase MRCK beta, EH domain-containing protein 2, Disintegrin and metalloproteinase domain-containing protein 10, EH domain-containing protein 4, and Calpain-2 catalytic subunit as the upregulated proteins. Conversely, the downregulated proteins consist of Nectin-2, Galactose-specific lectin nattectin, Interleukin-6 receptor subunit beta, Receptor-type tyrosine-protein phosphatase F, L-rhamnose-binding lectin CSL2, and Stomatin. These findings pave the way for further investigations into the potential utility of these significantly altered plasma membrane proteins as promising noninvasive biomarkers for in vitro diagnostics.

### 3.6. Differentially Expressed Proteins Interaction Analysis

To explore potential protein–protein interactions among the proteins carried by mucus vesicles upon entry into recipient cells, we performed an analysis using the StringDB protein interaction database. The analysis identified interactions among a total of 49 proteins, including 31 upregulated and 18 downregulated proteins (Supplementary Figure S3). Significantly, Protein_20640 (CAD protein), Protein_4007 (Proto-oncogene tyrosine-protein kinase Src), and Protein_14430 (CAD protein) stood out as central hub proteins within this network. Importantly, all three proteins exhibited significant upregulation following bacterial infection, suggesting their pivotal roles in the process of pathogen clearance. The CAD protein, referring to Caspase-Activated Deoxyribonuclease, acts as a nuclease responsible for inducing DNA fragmentation and chromatin condensation during apoptosis, facilitating the removal of infected cells. Similarly, Proto-oncogene tyrosine-protein kinase Src, encoded by the SRC gene, is a non-receptor protein tyrosine kinase activated by various cellular receptors, including immune response receptors, integrins, receptor protein tyrosine kinases, G-protein coupled receptors, and cytokine receptors. Src kinase participates in signaling pathways regulating a range of biological activities, such as gene transcription, immune response, cell adhesion, cell cycle progression, apoptosis, migration, and transformation. These findings suggest that mucus vesicles contribute not only to the clearance of extracellular surface pathogens but also possess complex mechanisms for pathogen removal in infected skin cells. Further investigations will help unravel the comprehensive processes underlying this intricate interplay.

### 3.7. Validation of DEP Genes

**Figure 7:**
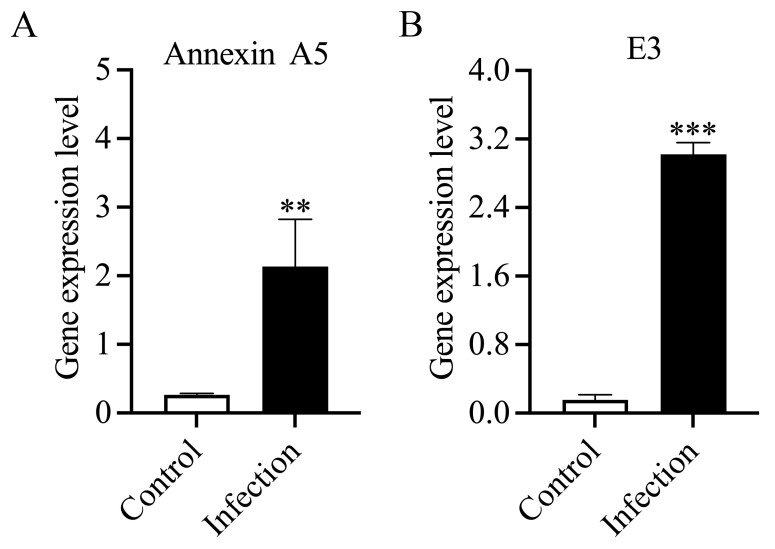
Validation of DEP genes in the control and *P. plecoglossicida* infection groups via qRT-PCR. (**A**) Annexin A5 expression level after *P. plecoglossicida* infection; (**B**) E 3 subunit M expression level after *P. plecoglossicida* infection. Annexin A5 and E 3 subunit M, were expressed at significantly higher levels in infected fish than in control fish and are considered potential biomarkers of *P. plecoglossicida* infection. ** and *** indicate the significantly expression level compare to the control group.

Quantitative RT-PCR was conducted to validate the expression of DEP genes. Annexin A5 (Figure 7A) and E 3 subunit M (Figure 7B) showed significantly higher expression in infected fish than in the control group. The results were consistent with the results of proteomic analysis and suggested the accuracy of the proteomic sequencing. However, these proteins (Annexin A5, E 3 subunit M) were considered potential biomarkers of *P. plecoglossicida* infected groupers.

## 4. Discussion

Skin mucus is a unique feature of aquatic animals, acting as the first line of defense against adverse environmental factors [17] and containing specific biomolecules crucial for symbiosis, mucosal immune system development, and pathogen invasion mechanisms [40]. Alterations in its molecular components can serve as indicative markers for evaluating pathological processes in adverse conditions [41]. Owing to its noninvasive nature and easy obtainability, mucus has natural advantages for noninvasive diagnosis and physiological state detection [42,43,44].

Exosomes, vesicles involved in intercellular communication and secreted from various cells, contain important biological molecules such as proteins and nucleic acids [45]. The presence of exosomes in fish skin mucus opens new possibilities for understanding intercellular communication and developing potential biomarkers for bacterial infection and immunological research. Mucus sampling for exosomes is noninvasive and holds promise for clinical and research applications, surpassing invasive approaches [45].

In this study, we successfully extracted exosomes from grouper skin mucus. These exosomes exhibited characteristic size and shape features, and the presence of the CD63, CD81, GAPDH, HSP70, and TSG101 proteins was confirmed through Western blotting. Furthermore, our proteomic analysis identified proteins enriched in the vesicle-mediated transport pathway, supporting the successful characterization of these exosomes.

After infection, we identified significantly altered plasma membrane proteins in mucus exosomes. We detected upregulated proteins, including catenin alpha-1, annexin A13, coatomer subunit beta, serine/threonine-protein kinase MRCK beta, EH domain-containing protein 2, disintegrin and metalloproteinase domain-containing protein 10, EH domain-containing protein 4, and the calpain-2 catalytic subunit. Conversely, the downregulated proteins consisted of Nectin-2, galactose-specific lectin nattectin, interleukin-6 receptor subunit beta, receptor-type tyrosine-protein phosphatase F, L-rhamnose-binding lectin CSL2, and Stomatin. These findings suggest the potential utilization of these significantly altered plasma membrane proteins as noninvasive biomarkers for in vitro diagnostics.

By comparing our results with those of the proteomic analysis of mucosal exosomes from *Vibrio harveyi*-infected *Cynoglossus semilaevis* by Zhao et al. [14,15], we detected differences in signature proteins. Zhao et al. [14] identified ferritin, TLR5S X1, calcium-transporting ATPase, histone H2B, and eIF5A as signature proteins in mucus exosomes, suggesting their potential as infection-related molecular biomarkers for *C. semilaevis*. Similarly, Zhao et al. [15] proposed aminopeptidase (Anpep), calcium-transporting ATPase (LOC103382755), histone H2B (LOC103381126), and histone H2A (LOC103381127) as candidate biomarkers in mucus, potentially replacing exosomes as a pool for bacterium-infected markers in *C. semilaevis*. Interestingly, in contrast to our findings, these proteins did not exhibit significant changes in the present study. These findings indicate that the use of mucous exosomes as noninvasive diagnostic targets for bacterial infections may result in high species specificity, thereby enhancing the prospects and significance of developing noninvasive diagnostic techniques based on mucous exosomes for aquatic animals.

The immune system of hosts has evolved complex mechanisms to limit the access of pathogenic microorganisms to essential micronutrients, including iron, zinc, and manganese, as part of a process known as nutritional immunity [46]. Nutritional immunity plays a pivotal role in controlling bacterial infections, particularly those dependent on iron acquisition [47]. Extensive research has also shed light on the importance of other transition metals, such as zinc, manganese, and copper, in nutritional immunity [48]. Deletion of the high-affinity zinc import system in bacterial pathogens has been demonstrated to attenuate their virulence in animal models [47]. Ferritin, an upregulated protein identified in the mucus exosomes of *C. semilaevis* following infection [14], responds to bacterial infection and inflammation by increasing its expression. As a critical iron storage protein, ferritin reduces serum iron levels to suppress bacterial growth. Its expression exhibits tissue-specific patterns, with pronounced levels observed in the liver, brain, and spleen during infection [49,50,51,52]. Studies on Antarctic notothenioid fish infected with *Piscirickettsia salmonis* have highlighted the essential role of ferritin in the infectious process, with its expression varying depending on the bacterial strain and tissue type [50]. The upregulation of ferritin has been reported in the liver during *Edwardsiella tarda* infection in Japanese flounder (*Paralichthys olivaceus*), in the spleen during *Aeromonas salmonicida* subsp. Salmonicida infection in rainbow trout (*Oncorhynchus mykiss*), and in the spleen during *Aeromonas hydrophila* challenge in blunt snout bream (*Megalobrama amblycephala*) [49,51]. Additionally, ferritin has been implicated in innate immune suppression in seabass larvae, acting by negatively regulating CXCR4 and leading to the inhibition of cell proliferation, differentiation, and migration during *Vibrio anguillarum* infection [52]. Our study confirmed the presence of ferritin in mucus exosomes from grouper, suggesting its potential role in nutritional immunity. Moreover, we identified other metal ion-binding proteins, such as the copper transport protein ATOX1 and zinc-binding protein A33, in mucus exosomes. However, their expression levels did not significantly change after infection in our study. This discrepancy could be attributed to the method of infection used, which involved injection and might have resulted in a lower pathogen concentration on the fish skin surface. Collectively, these findings support the concept of nutritional immunity within fish mucus exosomes, but further investigation is needed to fully understand the underlying mechanisms involved.

The balance between oxidative and reducing reactions is crucial for maintaining redox homeostasis in living systems. Our observation of the differential expression of proteins involved in oxidative-reduction reactions suggests that mucus exosomes may activate these reactions to eliminate surface pathogens and prevent further infection. However, this finding contrasts with previous studies on fish mucus exosomes [14,15], indicating potential high species specificity when mucus exosomes are used as noninvasive diagnostic targets for bacterial infections. Furthermore, we detected the presence of nuclear proteins, particularly histones, in mucus exosomes, which is consistent with findings from other studies. Histones have also been identified in the sweat exosomes of healthy individuals, where they are related to the barrier function of the skin [53]. These nuclear proteins may play a role in survival mechanisms by packaging harmful DNA into exosomes to maintain cellular homeostasis [54]. In fish, histones have been isolated and characterized mainly from the skin, head–kidney, and gonads. Their expression is modulated by infections or immune stimuli, such as viral or bacterial pathogens (e.g., NNV, *V. anguillarum*, and *Photobacterium damselae*), parasites (*Cryptocaryon irritans*), and PAMP or mitogen stimulation [55,56]. Histones have been confirmed to be involved in immune regulation in fish; however, their potential as candidate infection biomarkers has not been previously discussed.

In this study, we investigated the role of mucus exosomes in the immune response of grouper, with a particular focus on their potential as noninvasive diagnostic targets for bacterial infections. Our findings shed light on several key aspects related to the molecular composition and functionality of mucus exosomes, along with their implications for nutritional immunity and immune regulation. However, it is important to acknowledge the limitations of our study. First, the observed differential expression of proteins involved in oxidative-reduction reactions and the presence of histones in mucus exosomes require further in-depth investigation to fully understand their functional significance. Additional studies are needed to elucidate the specific mechanisms by which these proteins contribute to immune responses and bacterial infection control. Furthermore, the species specificity observed in the differential protein expression underscores the need for careful consideration when mucus exosomes are used as diagnostic targets. The high variability between species emphasizes the importance of species-specific studies to ensure accurate and reliable diagnostic techniques.

## 5. Conclusions

In conclusion, our study provides valuable insights into the role of mucus exosomes in immune responses, specifically in relation to oxidative-reduction reactions and the presence of histones. These findings expand our understanding of the molecular complexity of mucus exosomes and their potential in noninvasive diagnostics for bacterial infections. Further investigation into the functional significance of these proteins and the species-specific nature of mucus exosomes will increase our ability to develop effective diagnostic tools and advance our understanding of host–microorganism interactions in aquatic environments.

## Figures and Tables

**Table 1:** Primers used in present study.

Primers	Sequences (5′-3′)	Reference
β-actin-F	GTGCTGTCTTTCCCTCCATC	[39]
β-actin-R	CTCTTGCTCTGGGCTTCATC	
Grouper-Annexin A5-F	GAGGCAGGAGATCAAGACCG	
Grouper-Annexin A5-R	GAGCCACGATCAGGGTTTCA	
Grouper-E 3 subunit M-F	TGTCCGTGCTCTCAAAGACC	
Grouper-E 3 subunit M-R	TGCTAGCTTTCCGCTCACAA	

**Table 2:** Differentially expressed proteins (Top 20 upregulated and downregulated proteins).

	Protein	Description	log2FC	*p* Value	Subcellular Localization
1	Protein_21745	Plasma kallikrein	8.26	0.001	extracell protein
2	Protein_8061	Annexin A5	7.60	0.003	extracell protein
3	Protein_2146	Eukaryotic translation initiation factor 3 subunit M	7.40	0.005	--
4	Protein_11510	S-methyl-5-thioadenosine phosphorylase	7.35	0.005	cytoplasm protein
5	Protein_7712	Collagen alpha-1(XIV) chain	7.12	0.007	extracell protein
**6**	**Protein_8704**	**Catenin alpha-1**	**7.09**	**0.007**	**plasma membrane protein**
7	Protein_10555	60S ribosomal protein L17	7.06	0.008	--
8	Protein_21908	26S proteasome regulatory subunit 6A	7.01	0.008	nucleus protein
9	Protein_509	Actin, muscle	6.99	0.009	centrosome protein
**10**	**Protein_3526**	**Annexin A13**	**6.77**	**0.012**	**plasma membrane protein**
11	Protein_21718	FACT complex subunit SSRP1	6.75	0.012	nucleus protein
12	Protein_4455	Major vault protein (Fragment)	6.72	0.013	nucleus protein
13	Protein_4749	Calpain-5	6.64	0.014	cytoplasm protein
14	Protein_3222	Nck-associated protein 1	6.63	0.014	--
15	Protein_921	Extended synaptotagmin-1	6.58	0.015	microsome protein
**16**	**Protein_1062**	**Coatomer subunit beta**	**6.54**	**0.016**	**plasma membrane protein**
17	Protein_4013	Sodium/potassium-transporting ATPase subunit alpha-3	6.53	0.016	nucleus protein
18	Protein_20191	Myosin-10	6.52	0.017	nucleus protein
19	Protein_5803	Desmoplakin	6.48	0.018	--
20	Protein_6704	Synaptotagmin-like protein 4	6.42	0.019	cytoplasm protein
**21**	**Protein_1348**	**Nectin-2**	**−1.03**	**0.040**	**plasma membrane protein**
22	Protein_3796	COMM domain-containing protein 6	−1.06	0.039	nucleus protein
**23**	**Protein_1699**	**Galactose-specific lectin nattectin**	**−1.07**	**0.038**	**plasma membrane protein**
24	Protein_19641	ATP-dependent translocase ABCB1	−1.07	0.038	mitochondrion protein
25	Protein_10149	Serine/threonine-protein kinase Nek9	−1.14	0.035	cytoplasm protein
26	Protein_22073	RNA-binding protein 8A	−1.18	0.033	nucleus protein
27	Protein_13899	Transmembrane emp24 domain-containing protein 1	−1.19	0.033	Golgi apparatus protein
28	Protein_6507	Aldehyde dehydrogenase family 3 member B1	−1.20	0.033	endoplasmic reticulum protein
29	Protein_20110	N-alpha-acetyltransferase 15, NatA auxiliary subunit	−1.31	0.029	cytoskeleton protein
30	Protein_4676	Low choriolytic enzyme	−1.33	0.028	--
31	Protein_11132	Plectin	−1.40	0.026	cytoskeleton protein
32	Protein_6024	Plasminogen activator inhibitor 1 RNA-binding protein	−1.40	0.026	cytoplasm protein
**33**	Protein_8938	Cyclin-dependent kinase 4 inhibitor D	−1.42	0.025	nucleus protein
34	Protein_3505	Superoxide dismutase (Mn), mitochondrial	−1.43	0.025	mitochondrion protein
**35**	**Protein_10177**	**Galactose-specific lectin nattectin**	**−1.44**	**0.025**	**plasma membrane protein**
**36**	**Protein_10019**	**Interleukin-6 receptor subunit beta**	**−1.49**	**0.023**	**plasma membrane protein**
**37**	**Protein_2270**	**Receptor-type tyrosine-protein phosphatase F**	**−1.53**	**0.022**	**plasma membrane protein**
38	Protein_20694	NHL repeat-containing protein 3	−1.54	0.022	extracell protein
39	Protein_3470	Carbonyl reductase (NADPH) 1	−1.56	0.021	cytoplasm protein
40	Protein_431	E3 ubiquitin-protein ligase HECTD1	−1.58	0.021	endoplasmic reticulum protein

Note: Black bold letters indicate the potential surface biomarkers of mucus exosomes.

## Data Availability

All figures and tables used to support the results of this study have been included.

## Supplementary Materials

The following supporting information can be downloaded at: https://www.mdpi.com/article/10.3390/ani14233401/s1, Figure S1: GO enrichment analysis of DEPs; Figure S2: KEGG enrichment analysis of DEPs; Figure S3: Interaction analysis of DEPs; Table S1: Details of elution gradient; Table S2: List of all differentially expressed proteins.
