# Supplementary material for: Mucosal Exosome Proteomics of Hybrid Grouper Epinephelus fuscoguttatus♀ × E. lanceolatus♂ Infected by Pseudomonas plecoglossicida
Source: Animals (Basel). 2024 Nov 25;14(23):3401. doi: 10.3390/ani14233401 (PMC11640173; doi:10.3390/ani14233401)
Supplement: Supplementary file 1 [file animals-14-03401-s001.zip › Supplementary Table S1.pdf]

Supplementary Table 1. Details of elution gradient

Time (min)	mobile phase A (%)	mobile phase B (%)	Flow rate (mL/min)
0	97	3	1
10	95	5	1
30	80	20	1
48	60	40	1
50	50	50	1
53	30	70	1
54	0	100	1
