# Supplementary figures and images for: Mucosal Exosome Proteomics of Hybrid Grouper Epinephelus fuscoguttatus♀ × E. lanceolatus♂ Infected by Pseudomonas plecoglossicida
Source: Animals (Basel). 2024 Nov 25;14(23):3401. doi: 10.3390/ani14233401 (PMC11640173; doi:10.3390/ani14233401)

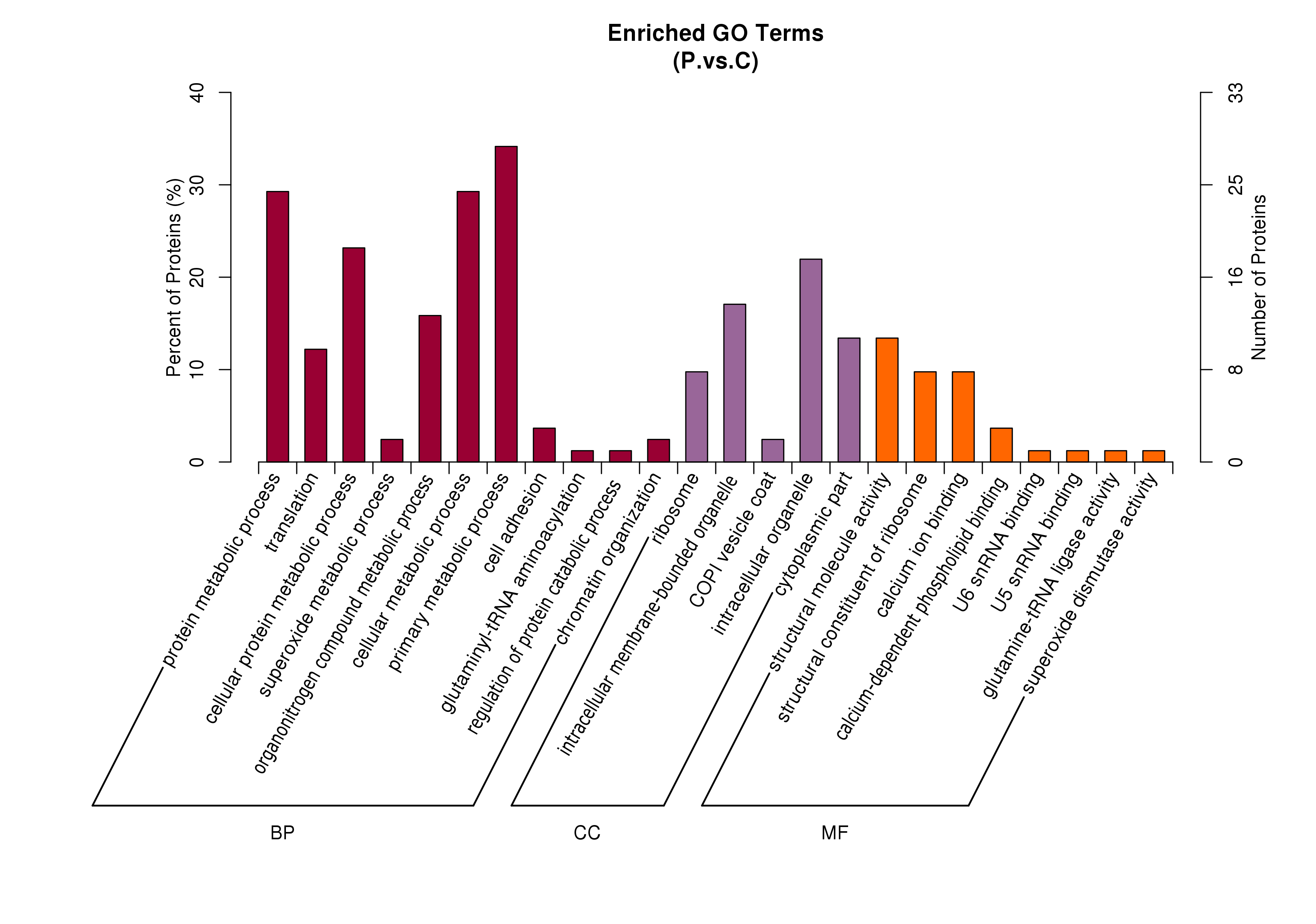

Supplement: Supplementary file 1 [file animals-14-03401-s001.zip › Supplementary Figure S1.tif]

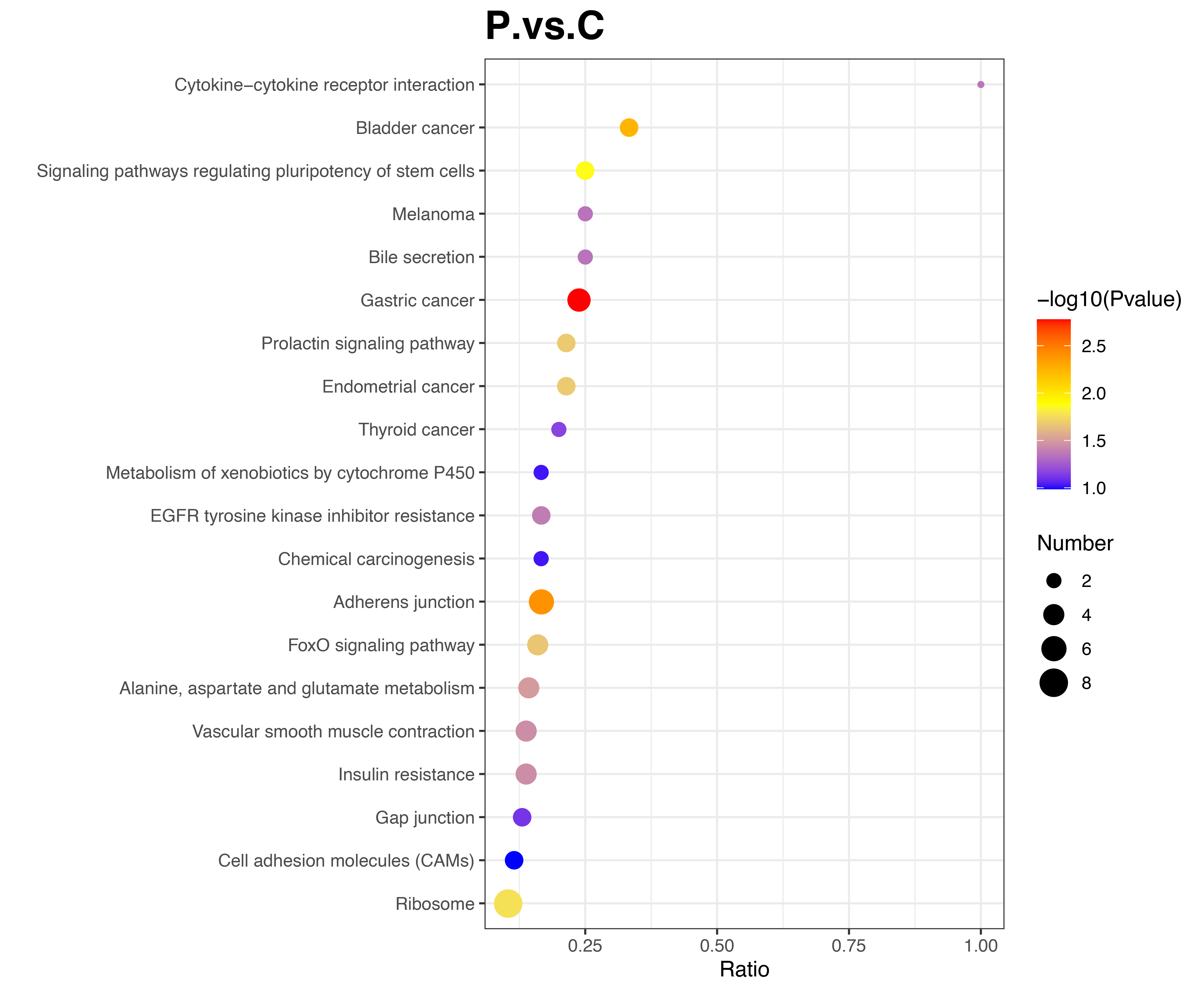

Supplement: Supplementary file 1 [file animals-14-03401-s001.zip › Supplementary Figure S2.tif]

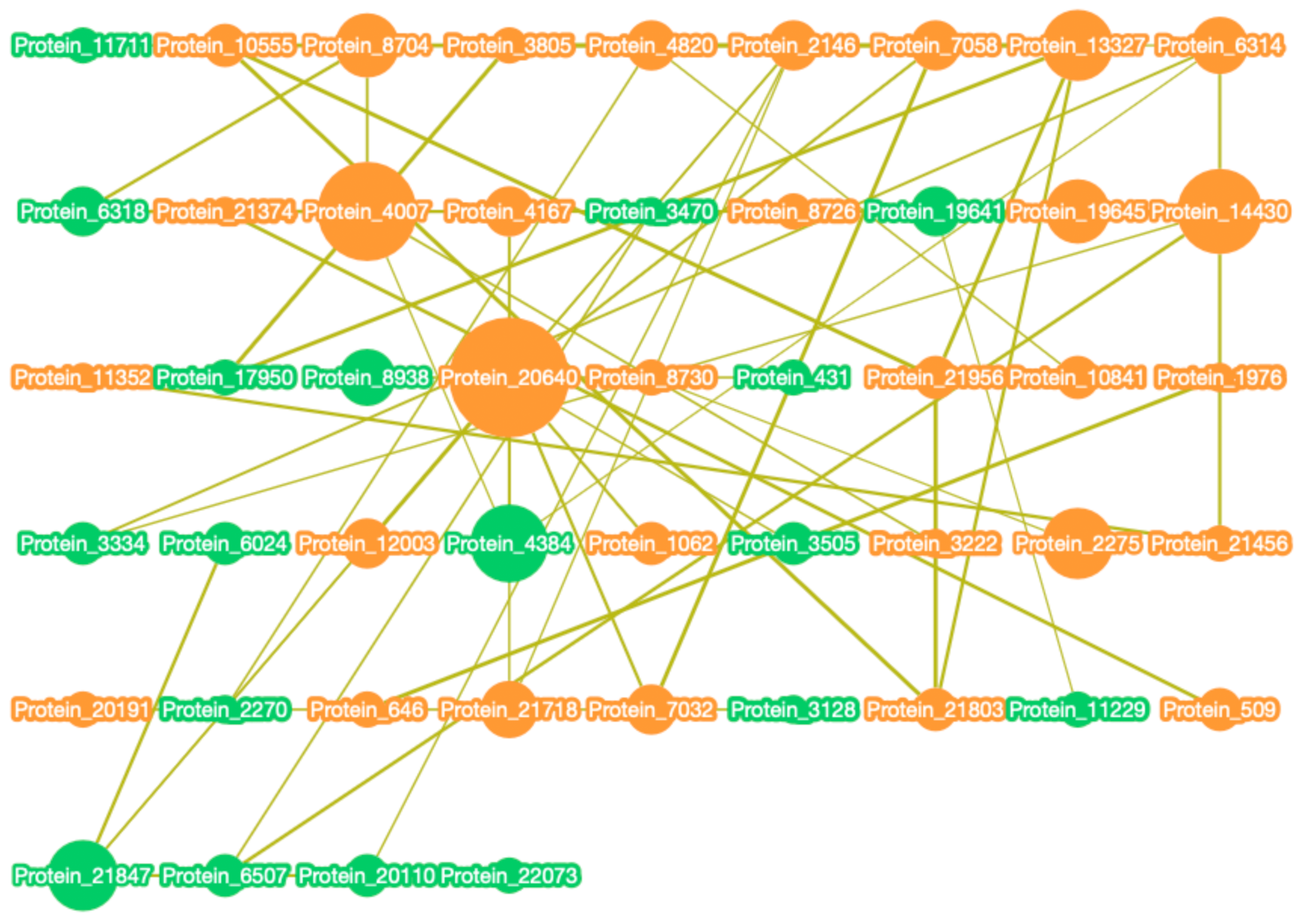

Supplement: Supplementary file 1 [file animals-14-03401-s001.zip › Supplementary Figure S3.tif]
